# Supplementary material for: Regulation of long-range BMP gradients and embryonic polarity by propagation of local calcium-firing activity
Source: Nat Commun. 2024 Feb 17;15:1463. doi: 10.1038/s41467-024-45772-4 (PMC10874436; doi:10.1038/s41467-024-45772-4)
Supplement: Supplementary file 1 — Supplementary Information [file 41467_2024_45772_MOESM1_ESM.pdf]

Supplementary information for

Regulation of long-range BMP gradients and embryonic polarity by propagation of local Calcium-firing activity

Hyung Chul Lee*, Nidia M.M. Oliveira, Cato Hastings, Peter Baillie-Benson, Adam A. Moverley, Hui-Chun Lu, Yi Zheng, Elise L. Wilby, Timothy T. Weil, Karen M. Page, Jianping Fu, Naomi Moris and Claudio D. Stern*

*Correspondence to: hyungchul@jnu.ac.kr, c.stern@ucl.ac.uk

This PDF file includes:

Supplementary Fig.1 to 10

Supplementary Tables 1 to 4

Supplementary Note 1

References for Supplementary information

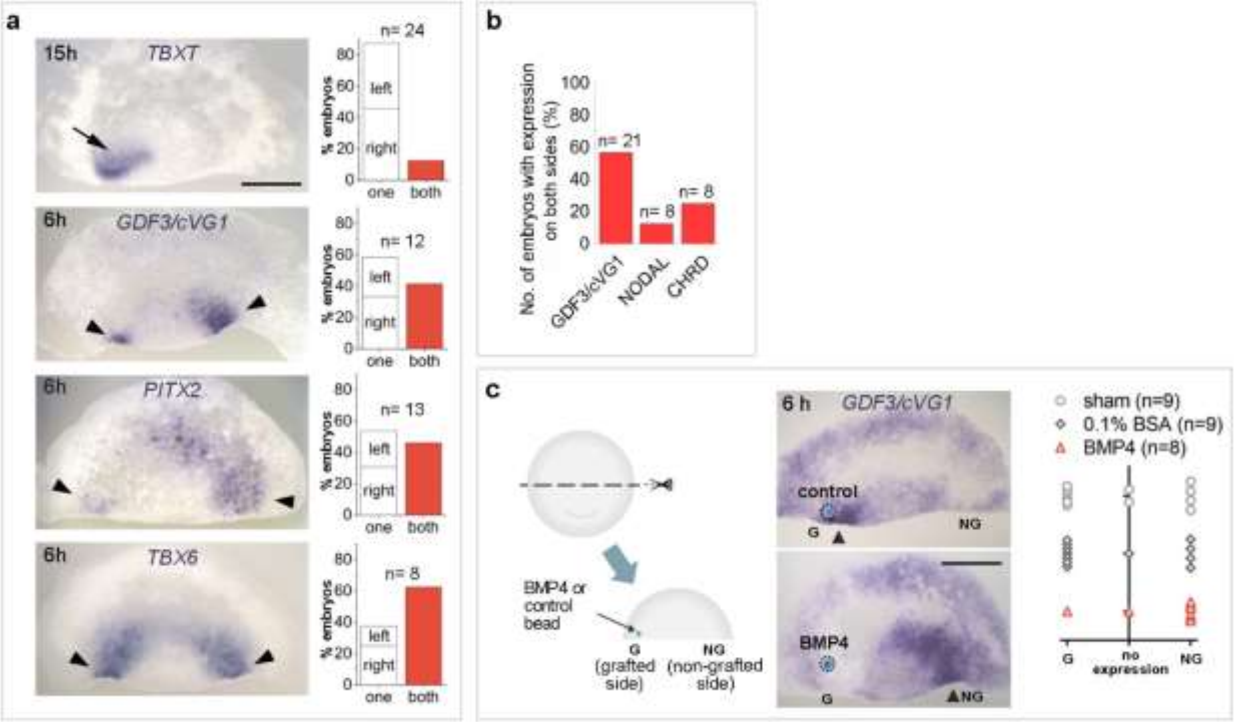

Supplementary Fig.1.

Long range competition for the site of gastrulation.

a. Formation of a primitive streak (arrow) expressing *TBXT* after 15 h culture, or expression of initial genes (*GDF3*, *PITX2*, and *TBX6*) (arrowheads) at 6 h culture in isolated anterior halves. A single primitive streak forms despite initiation (early marker expression) at both edges; this suggests long range communication and competition. Left: representative embryos. Right: quantification of anterior halves with either one-sided or two-sided (red) expression. Scale bar: 1mm. **b.** Quantification of anterior halves with expression on both sides either of an early gene (*GDF3*), or later genes (*NODAL* and *CHRD*) after 6-9 h culture[1]. The results suggest that communication starts at early stages after *GDF3* is expressed. **c.** *GDF3* expression after grafting nothing (sham), a control- or a BMP4-bead. Left: experimental design. Middle: representative embryos. Arrowheads indicate *GDF3* expression. Scale bar: 1mm. Right: quantification of the results, which suggest long range communication between the two extremes. Source data are provided as a Source Data file.

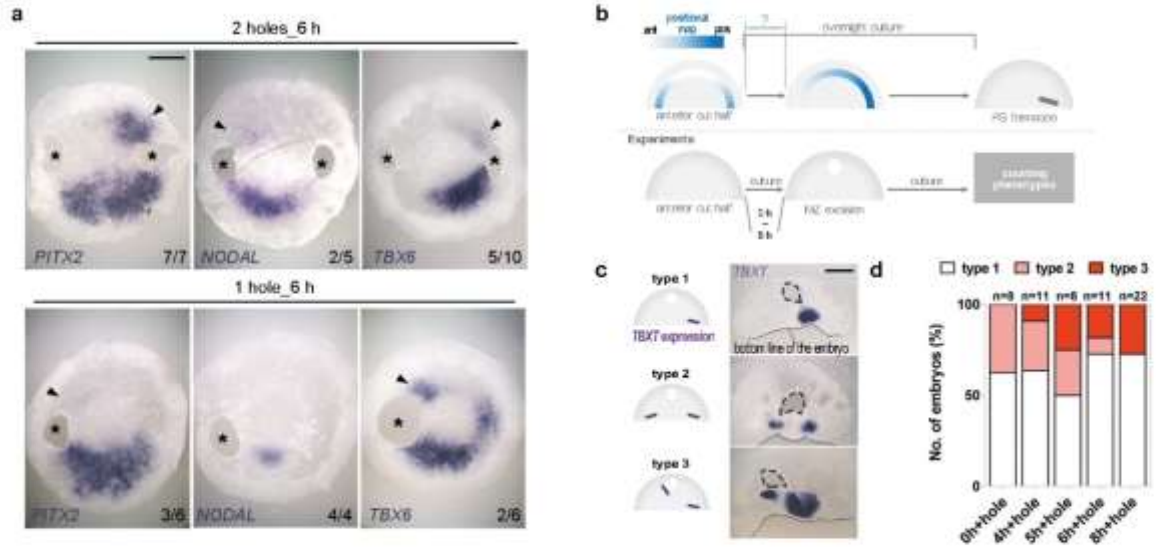

Supplementary Fig. 2.

The marginal zone as the route of communication of positional information in the embryo. **a.** Expression of initial genes in embryos with either two (top) or one (bottom) excisions in the marginal zone after 6 h culture. Embryos with one hole showed ectopic expression of *PITX2* and *TBX6*, but not *NODAL*, showing transient initial expression of early genes, despite this not resulting in PS formation after overnight culture as seen in Fig. 1 B. Asterisks: location of the excision. Arrowheads: ectopic gene expression. The number of embryos with ectopic expression is indicated. Scale bar: 1mm. **b.-d.** Experiment to estimate the speed of communication in the marginal zone. **b.** Hypothesis (top) and experimental design (bottom). **c.** Representative phenotypes. Dotted lines, location of the excision. Type 3 corresponds to repolarization of embryos in which the primitive streak developed from the anterior side. Scale bar: 1mm. **d.** Quantification of the different phenotypes observed. Source data are provided as a Source Data file.

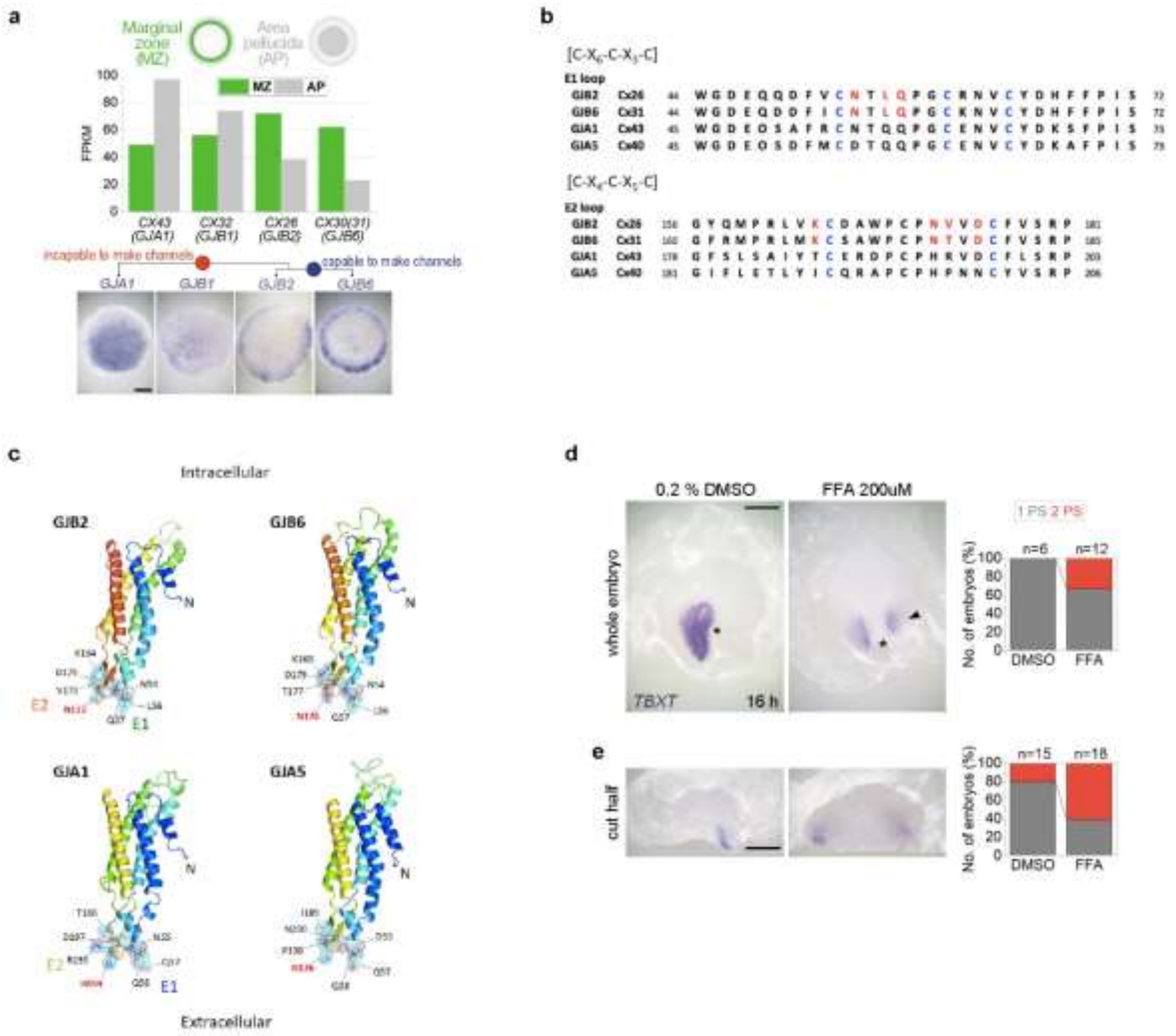

Supplementary Fig. 3.

Gap junctions as the route of communication in the marginal zone

a. Expression levels and spatial differences in the marginal zone and area pellucida of different connexins, based on RNAseq at pre-primitive-streak stages[2]. Bottom, mRNA in situ hybridization confirms the predictions of the RNAseq data. Scale bar: 1mm. **b.** Sequence alignment of selected chicken connexins E1 and E2 loops. Three cysteine residues (coloured blue) are highly conserved in both extracellular loops and were suggested to play an important role in stabilizing the structures during the docking of two opposing connexons[3], whereas the residues coloured in red are conserved among beta group connexins. Asparagine residues at position 172 and 176 of GJB2 and GJB6 respectively play a crucial role in forming hydrogen bonds at the docking interface with their opposing connexon from the same group, as previously reported[4]. **c.** Comparison between the modelled chicken alpha and beta group connexins. Conserved residues in the E1 and E2 loops of alpha and beta group connexins are labelled. Among these, the asparagine and histidine residues coloured in red from the E2 loop of the beta and alpha group respectively play a crucial role in

docking with a connexon from its own group. **d.-e.** Formation of a primitive streak after control (0.2 % DMSO) or flufenamic acid (FFA) treatment to whole embryos with excisions (asterisks) in the marginal zone (**d**) or in an isolated anterior half (**e**). Arrowhead: ectopic primitive streak. Scale bars: 1mm. Source data are provided as a Source Data file.

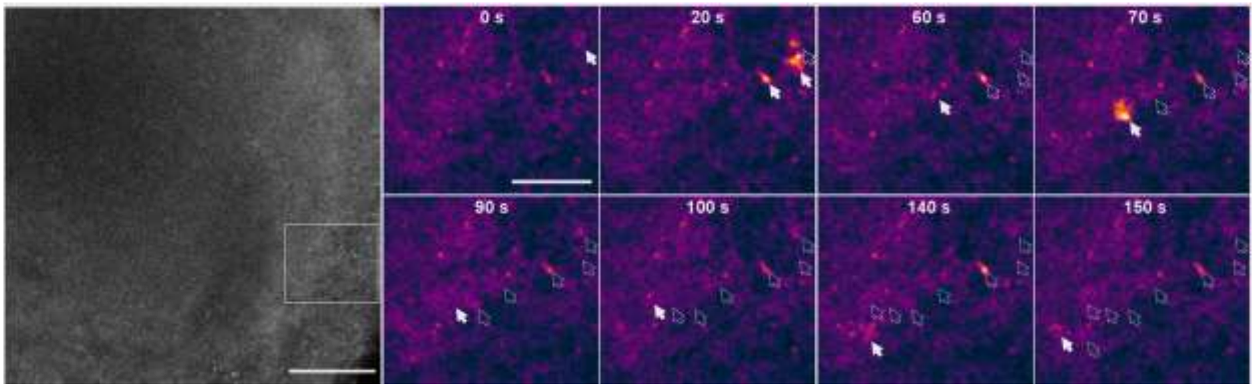

Supplementary Fig. 4.

Ca^{2+} signal analysis in an early primitive-streak stage embryo. Long travelling Ca^{2+} wave among cells in a group in the marginal zone (MZ) of an early primitive-streak stage embryo labelled with Cal520-AM (the same embryo at the bottom of Fig. 2A). The region marked with a square (left) is magnified (right). Scale bars: 500 µm (left) and 200 µm (right)**.** Filled and empty arrows indicate current and previous firings, respectively.

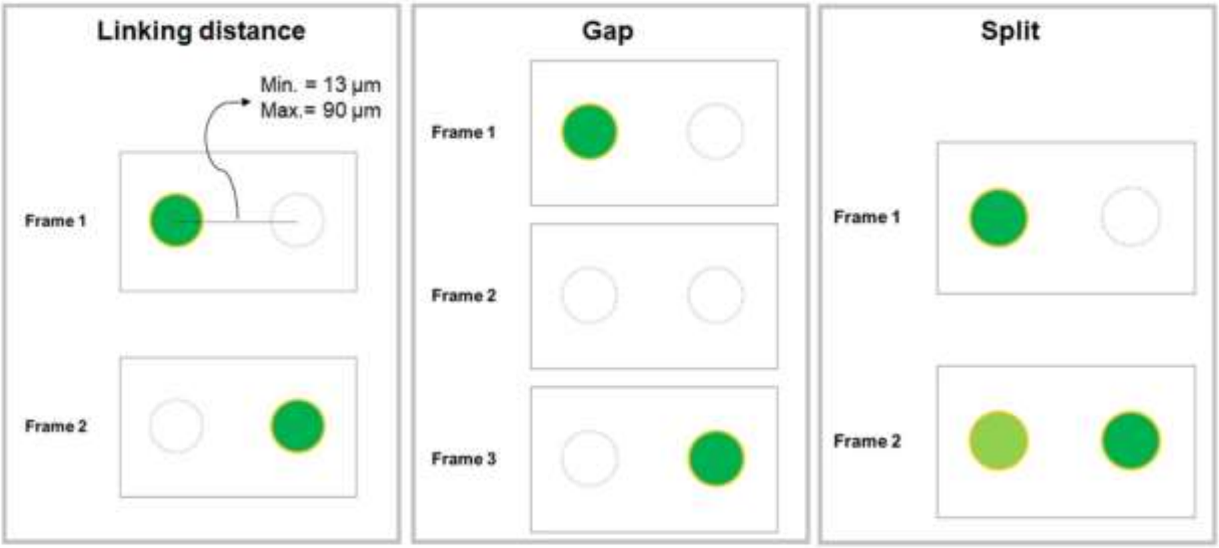

Supplementary Fig. 5.

Conditions to detect of Ca^{2+} tracks in different situation. LAP tracker in TrackMate plugin in Fiji was used to extrapolate Ca^{2+} tracks. Only tracks travelling over a distance >13 µm were considered, to exclude movements of single cells. The maximum distance of a wave between firing cells was set to 90 µm. A gap closing condition was implemented to include tracks with a gap between frames, while the splitting condition was used to detect tracks in those cases where the fluorescence of the first cell persists into the next frame.

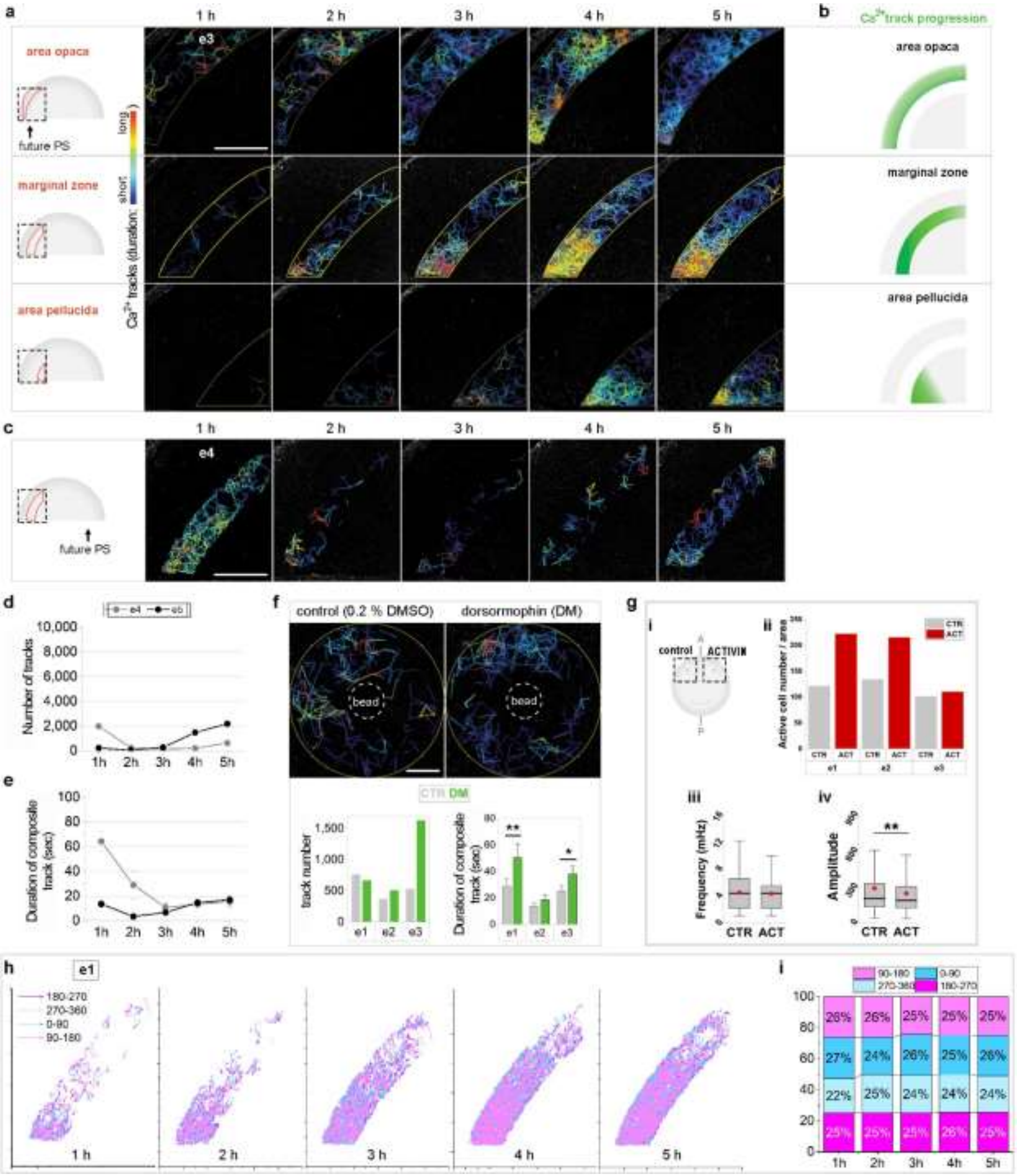

Supplementary Fig. 6.

Intercellular Ca^{2+} tracks and PS formation. a. Features of Ca^{2+} tracks in different embryonic regions (top to bottom) in the primitive-streak forming side of an isolated anterior half (e3, see Fig. 3). Diagrams on the left: dotted square, monitored region (with the analysed domain outlined in

red); e, embryo. The primitive-streak (PS)-forming side of the isolated anterior halves was monitored every hour for Ca^{2+} activity, and each region (area opaca, marginal zone and area pellucida) was analysed separately for Ca^{2+} tracks. Pseudo-colour coding represents track durations. *TBXT* expression was checked after overnight culture to decide the location of the PS-forming side. Dotted square: monitored region. Scale bar: 500 µm **b.** Schematic showing the overall direction of Ca^{2+} tracks in different regions. **c.-e.** Ca^{2+} tracks from non-PS-forming side of an embryo (e4) **(c)** and quantification of their number **(d)** and duration (e4 and e5) **(e)**. Details of the number of tracks are in the Methods. Scale bars: 500 µm. **f.** Ca^{2+} tracks analysis in the MZ of embryos after grafting a bead of dorsomorphin into the aMZ compared to controls (n = 3 embryos). Upper rows: representative figures showing Ca^{2+} tracks. Bottom rows: quantification of the number and duration of Ca^{2+} tracks. Values represent raw values or mean±s.e.m. Scale bar: 500 µm. Unpaired Student's *t*-test; ** P=0.00424, * P=0.01308. **g.** Ca^{2+} activity after control- or ACTIVIN-bead grafts in the aMZ. **gi:** experimental design. **gii:** active cell number (n=3 embryos). **giii** and **giv:** frequency and amplitude (relative fluorescent intensity) of the Ca^{2+} oscillations. n=357 and 547 cells for control and ACTIVIN, respectively. Unpaired Student's *t*-test; ** P=0.00609. In box plots, red dots and central lines indicate mean and median value. Box limits indicate the upper and lower quartiles, and whiskers show the range of values. **h.-i.** Angle distribution of the Ca^{2+} tracks of embryo e1 and their quantification. Vector maps showing the direction of the Ca^{2+} tracks (**g**) reveal a random distribution of angles (**h**). Source data are provided as a Source Data file.

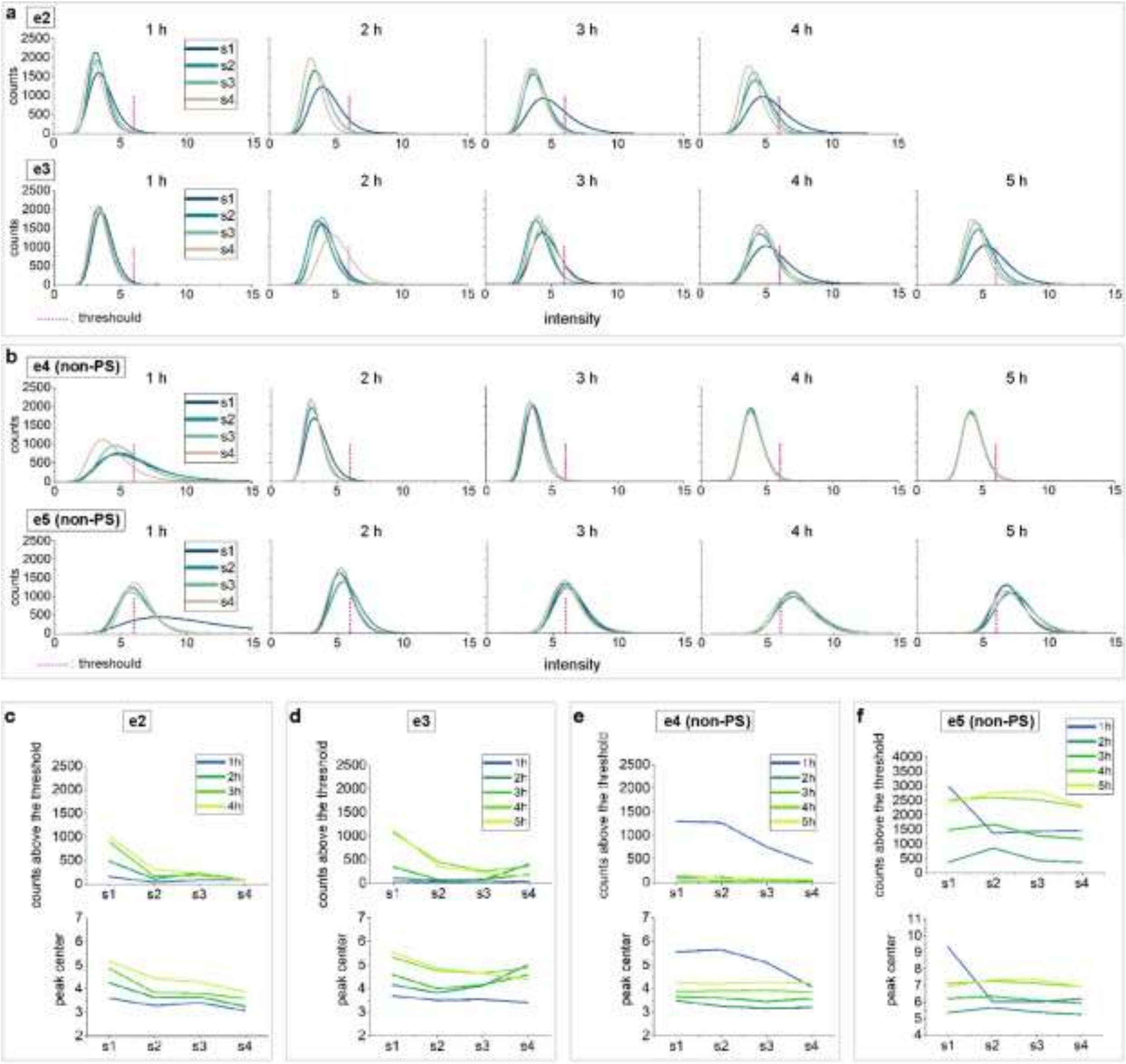

Supplementary Fig. 7.

Spatial and temporal changes in Ca^{2+} activity during initiation of primitive streak formation. The marginal zone either in the primitive-streak (PS)-forming side (e2 and e3; see Fig. 3 for e1) or in the non-PS forming side (e4 and e5) of several isolated half-blastoderms was monitored at every 1 h for analysis of regional difference in Ca^{2+} activity, over a period of 4-5 h. **a and b.** Histogram and comparison of four regions (s1-4 as in **Fig. 4b**) in e2 and e3 **(a)**, and e4 and e5 (non-PS region) **(b)**. x-axis: relative fluorescence intensity. y-axis: pixel counts. **c.-f.** Comparison in pixel counts above the threshold (magenta dotted line in **a and b**) and peak centre (the highest point in each graph) in e2 **(c)**, e3 **(d)**, e4 **(e)** and e5 **(f).** The threshold is set as 6, corresponding to ~10 % of the total counts at 1 h of e1 in Fig. 3f. Source data are provided as a Source Data file.

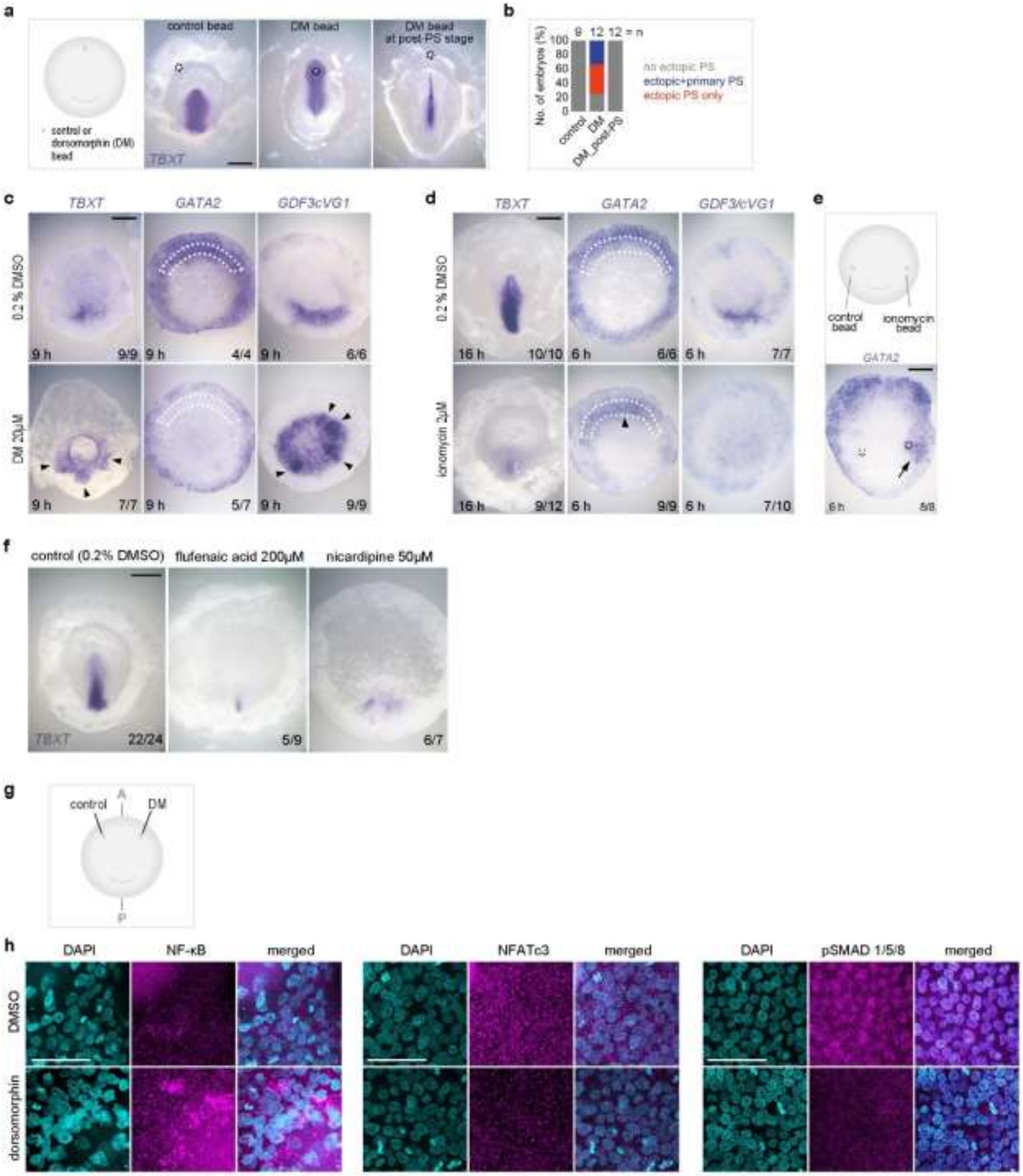

Supplementary Fig. 8.

BMP and Ca^{2+} activity regulate the position of the primitive streak. a. Ectopic PS formation after a control- (DMSO) or dorsomorphin (DM)-bead graft in the anterior marginal zone (aMZ). A DM-bead was grafted at pre-PS stage or after PS formation. Dotted circles: location of beads.

Scale bars: 1mm. **b.** Quantification of ectopic PS formation (with or without a primary PS). **c.** Effect of control (0.02 % DMSO) or DM treatment to the whole embryo on PS formation (*TBXT*), *GDF3*, and *GATA2* expression. The number of embryos similar to the represented phenotype is indicated: for *TBXT* and *GDF3/cVG1*, posterior (upper) or multiple (lower) expression; for *GATA2*, anterior (upper) or no (lower) expression. White dotted lines: aMZ. Arrowheads: multiple PS formation. Scale bars: 1mm. **d.** Effect of treatment with a control (0.1 % DMSO) or 2μM ionomycin of a whole embryo on PS formation (*TBXT*), *GDF3* and *GATA2* expression. The number of embryos similar to the represented phenotype is indicated: for *TBXT* and *GDF3/cVG1*, normal (upper) or decreased (lower) expression; for *GATA2*, normal (upper) or anteriorly localized (lower) expression. White dotted lines: aMZ. Arrowhead: localized *GATA2* expression in aMZ. Scale bars: 1mm. **e.** Upregulation of *GATA2* (black arrow) by an ionomycin-bead graft in the lateral MZ. Black dotted circle, location of beads. Incubation time and number of embryos with ectopic expression is indicated. Scale bars: 1mm. **f.** Effect of different chemicals on Ca^{2+} signal and PS formation. Representative embryos showing the PS (*TBXT*) after overnight culture with different chemicals. All chemicals have an inhibitory effect on PS formation. The number of embryos similar to the represented phenotype is indicated: for control, normal expression and primitive streak; for FFA and nicardipine, decreased expression and retarded primitive streak formation. Scale bars: 1mm. **g.-h.** Dorsomorphin regulates NF-κB, NFAT, and phospho-SMAD 1/5/9. **g.** Experimental design. **h.** A dorsomorphin-bead graft increases NF-κB expression near the bead compared to control, while it decreases expression of NFAT and nuclear localization of phospho-SMAD 1/5/8. A: anterior. P: posterior. Bright circles in the corner are the grafted beads. Scale bars: 50 μm. Source data are provided as a Source Data file.

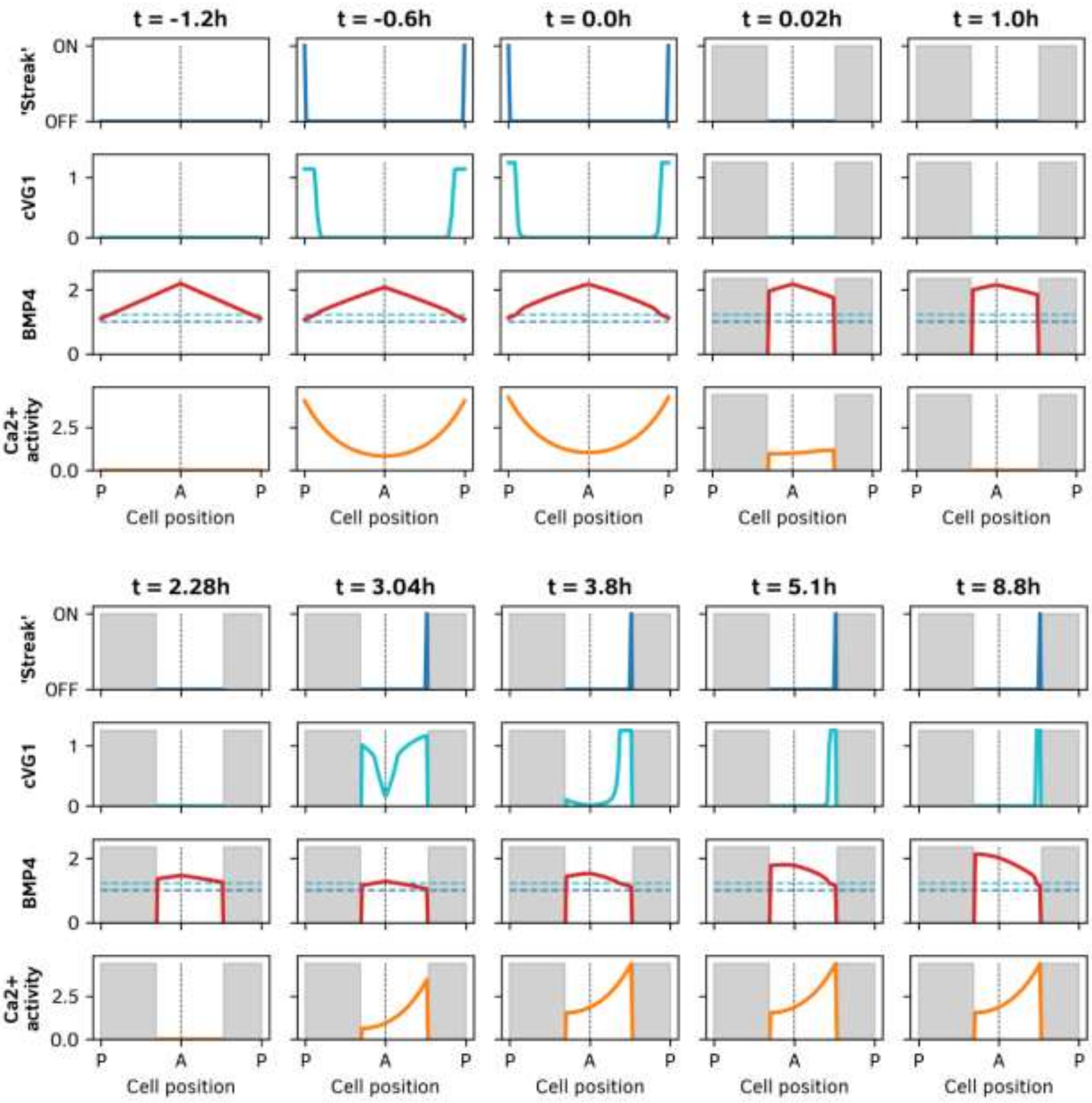

Supplementary Fig. 9.

Computer model of the consequences of asymmetric removal of the posterior portion of an embryo. Time shown relative to the removal of the posterior portion at t=0h. The x-axis represents cells of the marginal zone along the anterior (A)-posterior (P) axis. A and P: anterior and posterior extremes of embryo; greyed-out area: discarded embryo fragment. ‘Streak identity’ set to a binary state in each cell (ON/OFF). The level of calcium ‘activity’ is modelled, rather than concentration/amplitude/frequency of spikes. The simulation starts 1.2h before surgery with a shallow gradient of BMP4 decreasing anterior-to-posterior. GDF3/cVG1 and Ca^{2+}-activity are initially set to zero throughout. BMP4 levels decay and by t=-0.6 h, the level in the posterior part

is sufficiently low to induce cVG1 and for posterior cells to adopt ‘Streak identity’. This, in turn, triggers calcium activity posteriorly which quickly spreads throughout the embryo, forming a gradient opposite to that of BMP4. Before removal of the posterior half at t=0h, the embryo has almost reached steady state. At t=0.02h, the posterior part of the embryo has been removed with a slightly tilted cut (right side higher than left). 1h after the cut, the calcium activity level has decayed to zero, allowing BMP4 to decrease, as low calcium activity is required for BMP4 maintenance. At t=2.28h, BMP4 has fallen below threshold for inducing cVG1 on both left and right sides. By t=3.04h, BMP4 is low enough for a streak to be initiated on the right-hand side, also triggering a sharp increase in calcium. By t=3.8h calcium activity has spread throughout the anterior half and formed a gradient highest on the side of streak initiation. The high calcium activity increases BMP4 activity in a graded manner, repressing cVG1 production on the left side. BMP4 continues to be produced in graded manner corresponding to the calcium activity gradient. By t=8.8h, the BMP4 gradient has rotated to become parallel with the axis defined by the new streak on the right side.

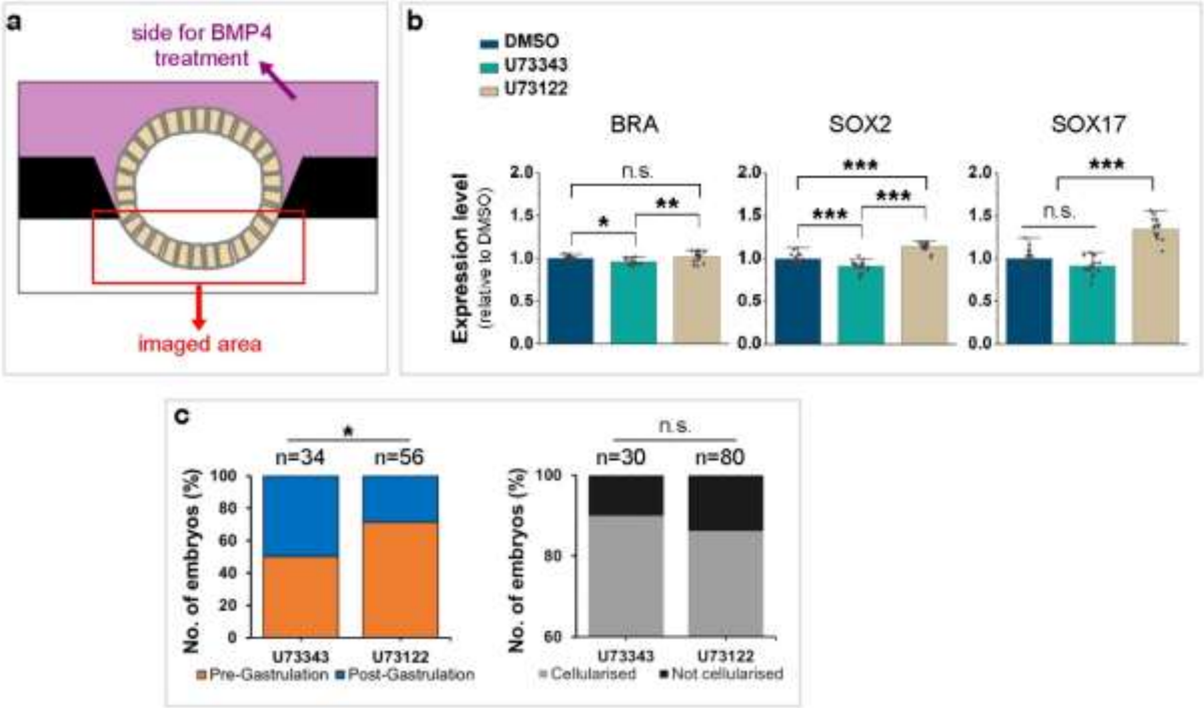

Supplementary Fig. 10.

A Ca^{2+} -based positioning system is conserved in vertebrates and invertebrates. a. Schematic diagram of the human microfluidic amniotic sac assay (μPASE) used for Calcium imaging (see Movie S7). **b.** Expression levels of BRA, SOX2 and SOX17 in human embryoids ("gastruloids") in suspension 1.5 h after chemical treatments with U73343 (control) or U73122 (PLC inhibitor) (n=16 for each). One-way ANOVA; *P* values for overall ANOVA are 0.0022, 2.4×10^{-13}, and 1.3×10^{-13}, respectively. Bonferroni correction for multiple comparisons. n.s.: not significant. **c.** Effect of the same chemicals on the initiation of gastrulation (left) and cellularization (right) in Drosophila embryos. Fisher's exact test; **P* =0.0462 (left). Source data are provided as a Source Data file.

Supplementary Table 1. Primitive streak formation after grafting two beads into the lateral marginal zone.

	**Two control-beads**		**Two ionomycin-beads**	
**phenotype**	**1 PS**	**2 PS**	**1 PS**	**2 PS**
**Trial 1**	13/13	0/13	7/9	2/9
**Trial 2**	6/6	0/6	9/13	4/13
**Trial 3**	8/8	0/8	9/12	3/12
**Total**	27/27	0/27	25/34	9/34

Supplementary Table 2. Parameters used for computational modelling.

**Type**	**Variable**	**Value**	**Units**
Thresholds	α	0.01	Arbitrary units (a.u.)
	β_C	1	ng mL^{-1}
	β_V	1.22	ng mL^{-1}
Production rates	k_B	1	ng mL^{-1} h^{-1}
	k_C	500	a.u. h^{-1}
	k_V	5	ng mL^{-1} h^{-1}
Decay rates	γ_0	0.3	h^{-1}
	γ_C	0.1	ng^{-1} mL h^{-1}
	γ_V	0.1	ng^{-1} mL h^{-1}
	λ	5	h^{-1}
	μ	4	h^{-1}
Propagation rate	D	50	mm^2 h^{-1}
Cell spacing	Δx	0.094248	mm

Supplementary Table 3. Results of varying parameters in computational model

Type	Symbol	Original Value	Varied values	Effect
Thresholds	α	0.01	0.001	Takes longer for BMP4 to decay below threshold, so slightly delays up initiation of cVg1 (approx. 0.5h) and streak formation.
			0.1	Takes less long for BMP4 to decay below threshold, so slightly speeds up initiation of cVg1 (approx. 0.7h) and streak formation.
	β_C	1	0.9	Broader domain of cVg1. Streak initiates later. Later streak initiation means that we cVg1 expression everywhere before resolving to one side.
			1.1	Streak initiates sooner. Narrower domain of cVg1 at equilibrium.
	β_V	1.22	*1.10	No cVg1 expression at equilibrium. β_V below critical threshold of 1.176.
			†1.18	Narrow domain of cVg1 at equilibrium.
			†1.46	Broader domain of cVg1 at equilibrium. Altered cVg1 dynamics after cut. cVg1 initiated earlier and across entire embryo.
Production rates	k_B	1	†0.851	Broader domain of cVg1 at equilibrium.
			†1.035	Narrow domain of cVg1 at equilibrium.
			*1.1	No cVg1 at equilibrium.
	k_C	500	*450	Less calcium activity throughout embryo. Narrow domain of cVg1. None at equilibrium.
			†465	Less calcium activity throughout embryo. Narrow domain of cVg1.
			†675	More calcium activity throughout embryo. Broader domain of cVg1.
	k_V	5	4.5	Lower cVg1 value at equilibrium giving narrower cVg1 domain.
			5.5	Higher cVg1 value at equilibrium giving broader

				cVg1 domain.
Decay rates	γ_0	0.3	0.27	Sped up dynamics after cut: cVg1 initiates earlier. Broader cVg1 domain.
			0.33	Delayed dynamics after cut: cVg1 initiates later. Narrower cVg1 domain.
	γ_C	0.1	*0.09	No cVg1 at equilibrium. Dynamics similar.
			†0.0928	Narrower domain of cVg1. Dynamics similar.
			†0.1352	Broader domain of cVg1. Dynamics similar.
	γ_V	0.1	0.09	Narrower domain of cVg1. Dynamics similar.
			0.11	Broader domain of cVg1. Dynamics similar.
	λ	5	4.5	Delayed dynamics. Narrower cVg1 domain.
			5.5	Sped up dynamics. Broader cVg1 domain.
	μ	4	3.6	Higher cVg1 value at equilibrium giving broader cVg1 domain.
			4.4	Lower cVg1 value at equilibrium giving narrower cVg1 domain.
Diffusion rate	D	50	45	Steeper gradient of calcium activity. Broader domain of cVg1 expression.
			55	Shallower gradient of calcium activity. Narrower domain of cVg1 expression.
Cell spacing	Δx	0.094248	Not varied	This parameter value has been chosen to ensure the appropriate size of the embryo, so that with 100 cells the circumference of the embryo is 9.42 mm, giving a diameter of 3 mm.

Supplementary Table 4. Results of varying initial conditions in computational model

Change in BMP4 gradient	Minimum (posterior) value	Maximum (anterior) value	Effect
Original	1.1	2.2	
Steeper	1.1	2.8	Reduces domain of cVg1 expression in the posterior. Delays onset of cVg1 expression after cut (by 0.22h from 2.38h to 2.60h).
Shallower	1.1	1.6	Broadens the domain of cVg1 expression in the posterior. Hastens onset of cVg1 expression after cut (by 0.24h from 2.38h to 2.14h).
Higher	1.3	2.4	Having the minimum BMP4 value above the threshold required to produce Vg1 means that there is no Vg1 expression initially. However, BMP4 quickly decays resulting in posterior Vg1 expression that we see in the pre-streak embryo.
*Lower	0.9	2.0	Starting with any cells below the calcium threshold is a problem. This is because more cells then stimulate calcium activity which floods the embryo, resulting in a streak everywhere.

Supplementary Note 1. Testing the robustness of the computational model: effects of varying parameter values

Overall, the model is robust to changes in parameter values. As expected for a biological, regulative system, when parameters are varied, there will be significant trade-offs between certain parameters. Explicitly, parameters are linked together so that when a change in the value of one parameter is made, the behaviour of the model can be maintained by a simultaneous change in another parameter. We can study these trade-offs by studying the behaviour of the model at equilibrium. So, if we consider what happens at equilibrium in an intact embryo, we set

$$\frac{\mathrm{d}B_i}{\mathrm{d}t} = \frac{\mathrm{d}C_i}{\mathrm{d}t} = \frac{\mathrm{d}V_i}{\mathrm{d}t} = 0\,. \qquad (1)$$

For cVg1, this then implies that where $\beta_V > B_i$, $V_i = {}^{k_V}\!/_{\mu} = 1.25$, and everywhere else $V_i = 0$. This implies that if you lower β_V, the effect can be nullified either by lowering k_V or increasing μ in turn.

If a cell has 'streak identity' ($A_i = 1$), it acts as a source of calcium activity which then spreads across the embryo. Then at equilibrium, the calcium activity manifests as a gradient decaying exponentially in space away from the source. By setting ${}^{\mathrm{d}C_i}\!/_{\mathrm{d}t} = 0$ and summing over i, we can derive that at equilibrium the total amount of calcium activity in the system will be equal to

$$\sum_{i=0}^{N} C_i = \frac{k_C N_A}{\lambda}\,, \qquad (2)$$

where N is the total number of cells and N_A is the total number of cells where $A_i = 1$. Therefore, the level of calcium activity is dependent on the parameters k_C and λ and the number of cells with 'streak identity'. The shape of the gradient of calcium activity is determined by the propagation rate D, where a higher value of D results in a shallower gradient and a lower value of D results in a steeper gradient. At equilibrium with the parameter values given in Supplementary Table 2, the minimum calcium value is 1.040 and the maximum value is 4.253 (3dp).

For BMP4, when the calcium activity is below threshold ($C_i < \alpha$), no BMP4 is produced and BMP4 will decay until for some i, $B_i > \beta_C$. Then the presence of calcium activity ($C_i > \alpha$) stimulates the production of BMP4 and when the system equilibrates, we can write

$$B_i = \frac{k_B}{\gamma_0 + \gamma_C C_i + \gamma_V V_i} . \quad (3)$$

Given that we know the equilibrium values for C_i and V_i, we can show that at the posterior side of the embryo where calcium activity and cVg1 are high, the equilibrium value for BMP4 is 1.176. The fact that this value is below the threshold for cVg1 production ($B_i < \beta_V = 1.22$) will ensure continued expression of cVg1 at the posterior side of the embryo. Changing any of the parameters (except α) will affect the equilibrium levels of BMP4, either directly (in the case of k_B, γ_0, γ_C, and γ_V) or indirectly by changing the equilibrium values of C_i and V_i. We can also derive that in the anterior side of the embryo, the equilibrium value for BMP4 will be 2.475.

To test the effects of varying parameter values, we first explored the effects of changing each value by 10% in each direction. In those cases where reducing or increasing the value by 10% caused the model to fail in some way (* in Supplementary Tables 3 and 4), we identified the full parameter range within which the model performs like the biological system. The results of this are shown as additional rows, marked † in Supplementary Table 3.

Parameter values that cause the model to fail result in either of two types of failure. The first is if the equilibrium value of BMP4 at the posterior side of the embryo is above the threshold to produce cVg1; no cVg1 expression is then seen. The second is if the overall level of calcium activity in the embryo increases significantly. This causes the level of BMP4 to fall, which means that more cells differentiate to 'streak identity', increasing the calcium activity still further. The result is that the entire embryo is flooded with calcium activity and all cells commit to 'streak identity'.

A real embryo has more than 100 cells. With a more realistic approximation of 748 cells, the model behaviour is identical. 3 parameters must be altered in turn: *Δ*x reduced by a factor of 7.48, k_C increased by a factor of 7.48, and *dt* (the time interval for the numerical solution) must be reduced by factor of 64, approximately 7.48^2. The reason this has not been done for every run is that reducing *dt* and increasing cell number, increases computation time by a factor of approximately 500.

We also tested the effect of replacing Heaviside functions with Hill functions (with a Hill coefficient of 4) for the production of BMP4 and for the production of cVg1. Overall, the behaviour of the model is comparable (Supplementary Movies 8 and 9).

In conclusion, in the model, as expected in a complex biological system that undergoes regulative behaviour, the embryo is able to compensate for variation in many parameters by altering others. This provides a robust homeostatic regulatory system that can ensure appropriate behaviour despite individual differences between embryos or environmental or other perturbations.

References

1 Skromne, I. & Stern, C. D. A hierarchy of gene expression accompanying induction of the primitive streak by Vg1 in the chick embryo. *Mech Dev* **114**, 115-118, doi:10.1016/s0925-4773(02)00034-5 (2002).

2 Lee, H. C. *et al.* Molecular anatomy of the pre-primitive-streak chick embryo. *Open Biol* **10**, 190299, doi:10.1098/rsob.190299 (2020).

3 Kumar, N. M. & Gilula, N. B. The gap junction communication channel. *Cell* **84**, 381-388, doi:10.1016/s0092-8674(00)81282-9 (1996).

4 Nakagawa, S. *et al.* Asparagine 175 of connexin32 is a critical residue for docking and forming functional heterotypic gap junction channels with connexin26. *J Biol Chem* **286**, 19672-19681, doi:10.1074/jbc.M110.204958 (2011).
