# Supplementary material for: Regulation of long-range BMP gradients and embryonic polarity by propagation of local calcium-firing activity
Source: Nat Commun. 2024 Feb 17;15:1463. doi: 10.1038/s41467-024-45772-4 (PMC10874436; doi:10.1038/s41467-024-45772-4)
Supplement: Supplementary file 3 — Description of Additional Supplementary Files [file 41467_2024_45772_MOESM3_ESM.pdf]

Description of Additional Supplementary Files

File Name: Supplementary Movie 1
Description: **cAMP activity in a live chick embryo.** cAMP imaging of a pre-PS-stage embryo labeled with Pink flamindo (cAMP indicator). Time is indicated in mm:ss.

File Name: Supplementary Movie 2
Description: **Ca^{2+} activity in a live pre-PS-stage chick embryo.** Ca^{2+} imaging of a pre-PS-stage embryo labeled with Cal-520 AM (Ca^{2+} indicator) showing spontaneous Ca^{2+} activity, which is particularly strong in the MZ. Left, bright field. Right, fluorescence. Pseudo-color coding represents fluorescence intensity. Time is indicated in mm:ss.

File Name: Supplementary Movie 3
Description: **Ca^{2+} activity in a live early primitive streak stage (HH stage 2) chick embryo.** Ca^{2+} imaging of an early streak stage embryo labeled with Cal-520 AM (Ca^{2+} indicator) showing spontaneous Ca^{2+} firing and particularly strong activity in the MZ. Left, bright field. Right, fluorescence. Pseudo-color coding represents fluorescence intensity. The marginal zone is marked by white lines is Time is indicated in mm:ss.

File Name: Supplementary Movie 4
Description: **Ca^{2+} activity appears to travel between non-adjacent cells via long cell protrusions.** Ca^{2+} imaging of the anterior marginal zone in an early-PS-stage embryo labeled with GCaMP6 by electroporation, showing firing along cell protrusions including lamellipodia and very thin filopodia. Time is indicated in mm:ss.

File Name: Supplementary Movie 5
Description: **Computer simulation of an intact embryo.** Details as in **Supplementary** Fig. 9.

File Name: Supplementary Movie 6
Description: **Computer simulation of an isolated anterior half-embryo, with a slightly tilted cut**. Details as in **Supplementary** Fig. 9.

File Name: Supplementary Movie 7
Description: **Live Ca^{2+} activity in microfluidic human amniotic sac embryoids.** Live Ca^{2+} imaging of the microfluidic human amniotic sac embryoids (μPASE) labeled with Cal-520 AM (Ca^{2+} indicator) without (left) or with BMP4 (right). The epiblast side of the embryoid is monitored (**Supplementary** Fig. 10a). Ca^{2+} activity is only seen in the BMP4-treated embryoid. Pseudo-color coding represents fluorescence intensity.

File Name: Supplementary Movie 8
Description: **Computer simulation of an intact embryo, where Hill functions have replaced Heaviside functions.**

File Name: Supplementary Movie 9
Description: **Computer simulation of an isolated anterior half-embryo, where Hill functions have replaced Heaviside functions**
